# Alcohol consumption patterns and HIV viral suppression among persons receiving HIV care in Florida: an observational study

**DOI:** 10.1186/s13722-017-0090-0

**Published:** 2017-09-27

**Authors:** R. L. Cook, Z. Zhou, N. E. Kelso-Chichetto, J. Janelle, J. P. Morano, C. Somboonwit, W. Carter, G. E. Ibanez, N. Ennis, C. L. Cook, R. A. Cohen, B. Brumback, K. Bryant

**Affiliations:** 1 Department of Epidemiology, College of Public Health and Health Professions and College of Medicine, University of Florida, PO Box 100231, Gainesville, FL 32610 USA; 2 Department of Medicine, University of Florida, Gainesville, FL USA; 3 Department of Clinical and Health Psychology, University of Florida, Gainesville, FL USA; 4 Department of Family, Community, and Health System Science, College of Nursing, University of Florida, Gainesville, FL USA; 5 Department of Biostatistics, University of Florida, Gainesville, FL USA; 6 Division of Infectious Diseases and International Medicine, University of South Florida Morsani College of Medicine, Tampa, FL USA; 7 Department of Internal Medicine, University of South Florida Morsani College of Medicine, Tampa, FL USA; 8 Florida Department of Health, Orange County Office of Clinical Services, Disease Investigation and Research, Orlando, FL USA; 9 Department of Epidemiology, Florida International University, Miami, FL USA; 10 National Institute on Alcohol Abuse and Alcoholism, Bethesda, MD USA

**Keywords:** Alcohol consumption, Binge drinking, HIV, Viral suppression, ART adherence

## Abstract

**Background:**

Alcohol consumption has been associated with poor antiretroviral therapy (ART) adherence but less is known about its relationship to HIV viral suppression, or whether certain drinking patterns have a stronger association than others. The objectives of this study were to determine the association of different patterns of alcohol consumption to HIV viral suppression and ART adherence, and to determine whether any associations of alcohol with HIV viral suppression were mediated by poor ART adherence.

**Methods:**

This observational study used baseline data from 619 HIV+ participants, recruited across 8 clinical and community settings across Florida as part of the Florida Cohort from 2014 to 2016. Alcohol consumption was measured by self-report, and grouped into four categories: heavy drinking (>7/week for women or >14 drinks/week for men); binge, but not heavy drinking (≥4 or >5 drinks/occasion for women and men, respectively), low level drinking (neither heavy nor binge), and abstinence. Serum HIV RNA measurements were obtained from statewide HIV surveillance data, and durable viral suppression was defined as achieving HIV viral suppression (<200 copies/ml) at every assessment in the past 12 months.

**Results:**

The majority of the 619 participants were male (63%) and aged 45 or greater (65%). The proportion of participants with heavy, binge, low-level drinking and abstinence was 9, 25, 37 and 30%, respectively. Optimal ART adherence (≥95%) was reported by 68%, and 60% achieved durable viral suppression. In multivariable analysis controlling for demographic factors, drug use, and homelessness, heavy drinking (compared to abstinence) was associated with increased odds of failing to achieve durable viral suppression (OR 2.16, 95% CI 1.08–4.32) whereas binge drinking alone was not significantly associated with this outcome (OR 1.04, 95% CI 0.64–1.70). Both heavy drinking and binge drinking were significantly associated with suboptimal ART adherence. Mediation analyses suggested that only a small proportion of the relationship between heavy drinking and suboptimal viral suppression was due to poor ART adherence.

**Conclusions:**

Exceeding weekly recommended levels of alcohol consumption (heavy drinking) was significantly associated with poor HIV viral suppression and ART non-adherence, while binge drinking was associated with suboptimal ART adherence in this sample. Clinicians should attempt to address heavy drinking in their patients with HIV.

## Background

Despite widespread availability of antiretroviral therapy (ART) in the United States, viral suppression (<200 copies/ml) is achieved in only 25% of all persons living with HIV and 70% of those retained in care [1]. Lack of HIV viral suppression is associated with more rapid HIV disease progression, cardiovascular complications [2] and transmission of HIV within the community [1]. Therefore, strategies to improve HIV viral suppression are an important part of the current national HIV strategy [3].

Alcohol consumption is common in persons with HIV infection [4] and has been linked to several adverse health outcomes in this population [5]. Alcohol consumption could affect HIV viral suppression via both behavioral (e.g. ART adherence) and biological (e.g. immune dysfunction) mechanisms [5]. While researchers have consistently found an association between alcohol consumption and poor ART adherence [6, 7], relatively few studies have reported on the association of alcohol consumption to HIV viral suppression in the ART era [8]. These studies have produced mixed results, with some showing an association of alcohol use with HIV disease progression and poor viral suppression [9–11], while others find no significant association [12, 13].

Some of the differences in findings across different populations could be due to the way that alcohol exposure was defined, or whether or not the investigators controlled for potential confounding variables. In general, papers focusing on alcohol use feature a wide range of definitions to define alcohol consumption, ranging from “any” versus “none”; quantity/frequency of drinking (e.g. number of drinks per week), binge drinking (e.g. number of drinks consumed in one drinking session), to the presence of an alcohol use disorder. Differences in these definitions can lead to inconsistent results that are difficult to compare by study [14].

Alcohol consumption often overlaps with several other health conditions or behaviors that could be linked to poor HIV viral suppression, including social determinants of health (e.g. homelessness) [15, 16], comorbid behavioral health conditions (e.g. depression and anxiety) [17, 18], and other substance use [17–20]. These overlapping factors should be considered when making a conclusion about whether alcohol consumption does have an independent effect on HIV viral suppression.

Little is known about which specific patterns of drinking are most strongly associated with poor HIV viral suppression, and whether any associations can be explained by the impact of alcohol on ART adherence. Therefore, the objectives of this study were to determine the association of alcohol consumption to HIV viral suppression using two different definitions of alcohol consumption patterns (heavy use and binge-drinking), and to determine whether any associations could be explained by differences in ART adherence.

## Methods

### Participants

Between October 2014 and December 2016, we recruited 903 participants living with HIV/AIDS into the Florida Cohort. Initiated in 2014 and ongoing, the Florida Cohort collects self-reported information about demographic and behavioral factors that may affect health outcomes for persons with HIV/AIDS. Participants were recruited from a collaborative network of county health departments and community setting clinics throughout Florida (includes sites at Lake City, Gainesville, Tampa, Orlando, Sanford, Ft. Lauderdale, and Miami). Any person with HIV greater than 18 years of age was eligible to participate in the study. After providing written informed consent, participants completed an anonymous, self-administered questionnaire examining demographics, substance use, mental health symptoms, and HIV-related health behavior. In order to ensure that all interested and eligible persons could participate, study staff asked if assistance was needed to complete the questionnaire; in such cases the questionnaire was read to the participants. The survey took approximately 30–45 min to complete, and participants received a $25 incentive for their time.

With approval from the Florida Department of Health, survey responses were securely linked to medical records to obtain additional data on anti-retroviral medications, co-morbid health conditions, and laboratory test results, including HIV viral load and CD4+ T-cell count. Florida mandates that HIV viral load test results are reported to the statewide HIV surveillance system; for this reason, the surveillance information was able to be matched and linked for 97% of all study participants. The research procedures are approved by the institutional review boards (IRB) at the University of Florida, Florida International University, and the Florida Department of Health. In order to study the effect of alcohol on durable viral suppression, we limited this analysis to 619 participants who had been diagnosed with HIV for at least 1 year, who had at least 2 HIV viral load test results in the prior 12 months, and who did not have missing data for alcohol exposure. The 619 persons included in the analysis were not significantly different from those excluded for missing data about alcohol consumption in terms of age, race, or gender.

### Measures

#### Alcohol consumption

Participants self-reported their alcohol consumption by answering the questions “In the past 12 months, how often did you have a drink containing alcohol” (options: less than 1–3 times a month, 1–3 times a week, 4–6 times a week, every day), “How many standard drinks would you have on a typical day” (range 0 to 6+), and “How often did you have 4+ standard drinks for women or 5+ standard drinks for men on one occasion? (options: never, less than monthly, monthly, weekly, or daily/almost daily)”. These three drinking items are based on the AUDIT-C, a 3-item scaled used to screen for drinking in primary care settings [21]. Using this information, plus the question “during the last 30 days, what is the largest number of drinks that you drank within a 24-h period”, we defined two different patterns of alcohol consumption. Average weekly consumption was determined by multiplying the average quantity and frequency, and “heavy drinking” was defined as consuming more than 7 or 14 drinks/week for women or men [22]. Binge drinking focused on the amount consumed in one sitting, and was defined as consuming ≥4 or ≥5 drinks for women or men at least once a month in the past 12 months [22]. Because over 90% of heavy drinkers also reported binge drinking, we categorized each person into one of four categories: Heavy (regardless of binge status); binge (not including heavy drinking), low level drinking (any drinking that was not heavy or binge), or no current drinking (abstinence).

#### Covariates

The study questionnaire assessed socio-demographic variables, including age, gender, race and ethnicity, education and homelessness. The Personal Health Questionnaire Depression Scale (PHQ-8 score) and Generalized Anxiety Disorder 7-item scale (GAD-7) were used to assess current depressive symptoms (PHQ-8 score ≥10) and current anxiety symptoms (GAD-7 score ≥10), respectively [23, 24]. Current tobacco use was categorized as yes or no. We created a dichotomous summary variable that indicated any use of injected or non-injected illicit drugs in the past 12-months, including marijuana, cocaine/crack, heroin, pain medications (like oxycontin), sedatives, methamphetamine, ecstasy, and other stimulants.

#### Antiretroviral (ART) adherence

ART adherence was assessed by self-report, as this has been shown to correlate strongly with HIV outcomes and is simpler and less costly to obtain compared to other ART adherence assessment options [25, 26]. ART adherence was defined as the proportion of days in last 30 days they did not miss any medications, based on their response to the question, “In the last 30 days, how many days did you miss at least one dose of any of your HIV medication?”, and was dichotomized as ≥95 versus <95% [27]. Adherence of 95% or greater is considered optimal adherence and has been associated with improved outcomes in terms of CD4 count and viral suppression [28, 29].

#### Viral suppression

HIV viral load values were obtained from HIV surveillance data. We considered the lab value closest to the time of baseline survey completion, and also all HIV viral load measures obtained up to 12 months prior to survey completion. We defined HIV viral suppression as an HIV-1 RNA test value ≤200 copies/ml. Durable viral suppression was achieved if all HIV viral load tests were suppressed viral load (≤200 copies/ml) in the past 12 months.

### Data analysis

We first conducted a descriptive analysis comparing potential risk factors with the four patterns of drinking (heavy, binge, low, and none), using the Chi square test to assess for statistical significance across the 4 drinking categories. We compared the proportions of participants achieving durable HIV viral suppression and ≥95% ART adherence in each of the 3 drinking patterns compared to non-drinkers. We then conducted multivariable logistic regression analyses to identify the relationship of the different drinking patterns with durable viral load suppression, after controlling for demographic and behavioral characteristics that were significantly associated with durable viral suppression in bivariate analysis (p < .10). Crude and adjusted odds ratios with 95% confidence limits were reported. To assess whether the relationship of alcohol consumption to viral suppression was mediated by ART adherence, we conducted a mediation analysis [30]. The mediation analysis sought to differentiate the direct effects of heavy drinking on HIV viral suppression from indirect effects mediated by ART adherence. Because the dependent variable was not rare, we used a generalized linear model regression with a log link function for viral suppression, which produces estimates of risk ratios. No covariates were included due to convergence issues when fitting a log-linear model for the outcome. The mediation macro developed by Valeri and VanderWeele was used to calculate the bootstrap confidence intervals with 1000 bootstrap samples [30].

Overall, missing data on most covariates of interests were minimal (<3%). However, approximately 9% of participants did not report or partially reported the use of illegal drugs in the past year. In order to address the potential for biased estimates, multiple imputation was performed using fully conditional specification (FSC) method to generate 20 complete datasets. All variables in the multivariable model were used in the imputation. All data analyses were performed in SAS version 9.4 (SAS Institute, Carry, NC, USA).

## Results

Characteristics of the 619 study participants are presented in Table 1. The majority of the sample was male (63%), aged 45 years or greater (65%), and diverse in terms of race/ethnicity and education status (Table 1). Homelessness in the past year was reported among 16%. Current smoking and illicit drug use was reported by 53 and 58%, respectively. Symptoms of depression (31%) and anxiety (29%) were relatively common. Durable viral load suppression was achieved among only 60% of the sample. Among those currently using ART (94%), 68% reported optimal ART adherence. Thirty percent of the sample reported no use of alcohol in the past year, 9% met criteria for heavy drinking (exceeds weekly limits), 25% met criteria for binge drinking (but not heavy drinking), and 37% reported low level, non-binge drinking.

**Table 1:** Baseline characteristics of persons living with HIV in the Florida Cohort (N = 619), 2014–2016

Baseline characteristics	No. (column %)
Gender	
Male	393 (63)
Female	226 (37)
Race	
Non-Hispanic White	137 (22)
Non-Hispanic Black	346 (56)
Hispanic	112 (18)
Other	24 (4)
Age group	
18–34	92 (15)
35–44	125 (20)
45–54	246 (40)
≥55	156 (25)
Education	
<High school	205 (33)
High school or equivalent	189 (31)
>High school	222 (36)
Homelessness	
No	513 (84)
Yes	96 (16)
Current smoking	
No	280 (47)
Yes	313 (53)
Any illicit drug use	
No	236 (42)
Yes	328 (58)
Current depressive symptoms	
No	416 (69)
Yes	188 (31)
Current anxiety symptoms	
No	428 (71)
Yes	174 (29)
Current ART use	
No	35 (6)
Yes	579 (94)
Current ART adherence	
<95%	177 (32)
≥95%	371 (68)
Durable viral load suppression	
≤200 copies/ml	369 (60)
>200 copies/ml	250 (40)
Drinking status	
Abstinence	184 (30)
Low, non-binge drinking	226 (37)
Binge drinking only	155 (25)
Heavy drinking	54 (9)

*ART* antiretroviral therapy

Descriptive analyses showing the distribution of potential covariates to the four drinking categories are shown in Table 2. Heavy and/or binge drinking were more common among those with less than a high-school education, those who smoked or used illicit drugs, persons who had been homeless in the past year, and persons with symptoms of depression or anxiety.

**Table 2:** Bivariate associations between covariates and alcohol consumption among the Florida Cohort (N = 619), 2014–2016

Characteristic	Drinking status (past year)					
	Total	N (Row %)				p value
		Abstinence	Low	Binge only	Heavy	
Gender						
Male	393 (63)	95 (24)	163 (41)	103 (26)	32 (8)	<.001
Female	226 (37)	89 (39)	63 (28)	52 (23)	22 (10)	
Race						
Non-Hispanic White	137 (22)	40 (29)	48 (35)	34 (25)	15 (11)	.95
Non-Hispanic Black	346 (56)	106 (31)	122 (35)	88 (25)	30 (9)	
Hispanic	112 (18)	30 (27)	47 (42)	27 (24)	8 (7)	
Other	24 (4)	8 (33)	9 (38)	6 (25)	1 (4)	
Age group						
18–34	92 (15)	17 (18)	41 (45)	26 (28)	8 (9)	.45
35–44	125 (20)	39 (31)	43 (34)	33 (26)	10 (8)	
45–54	246 (40)	79 (32)	83 (34)	59 (24)	25 (10)	
≥55	156 (25)	49 (31)	59 (38)	37 (24)	11 (7)	
Education						
<High school	205 (33)	69 (34)	50 (24)	57 (28)	29 (14)	<.001
High school or equivalent	189 (31)	52 (28)	77 (41)	48 (25)	12 (6)	
>High school	222 (36)	60 (27)	99 (45)	50 (33)	13 (6)	
Homelessness						
No	513 (84)	154 (30)	194 (38)	122 (24)	43 (8)	.08
Yes	96 (16)	23 (24)	29 (30)	33 (34)	11 (11)	
Current smoking						
No	280 (47)	97 (35)	112 (40)	58 (21)	13 (5)	<.001
Yes	313 (53)	77 (25)	105 (34)	92 (29)	39 (12)	
Any illicit drug use						
No	236 (42)	99 (42)	80 (34)	46 (19)	11 (5)	<.001
Yes	328 (58)	54 (16)	132 (40)	101 (31)	41 (13)	
Current depressive symptoms						
No	416 (69)	127 (31)	156 (38)	106 (25)	27 (6)	.08
Yes	188 (31)	53 (28)	63 (34)	48 (26)	24 (13)	
Current anxiety symptoms						
No	428 (71)	123 (29)	170 (40)	104 (24)	31 (7)	.04
Yes	174 (29)	54 (31)	50 (29)	49 (28)	21 (12)	
Current ART Adherence^a^						
<95%	177 (32)	32 (18)	64 (36)	59 (33)	22 (12)	<.001
≥95%	371 (68)	130 (35)	136 (37)	82 (22)	23 (6)	
Durable ART suppression						
≤200 copies/ml	369 (60)	121 (33)	135 (37)	91 (25)	22 (6)	.01
>200 copies/ml	250 (40)	63 (25)	91 (36)	64 (26)	32 (13)	

^a^Among participants who were currently taking ART. p values represent Chi square tests that the distribution of the variable varies by alcohol pattern

**Fig. 1:**
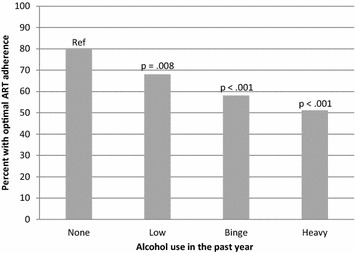
Proportion of 579 persons with HIV who are receiving ART that report optimal (≥95%) adherence to antiretroviral treatment in the previous month, according to alcohol consumption classification. Binge drinking defined as exceeding daily (but not weekly) drinking recommendations, and heavy drinking defined as exceeding weekly drinking recommendations. Florida Cohort, 2014–2016

**Fig. 2:**
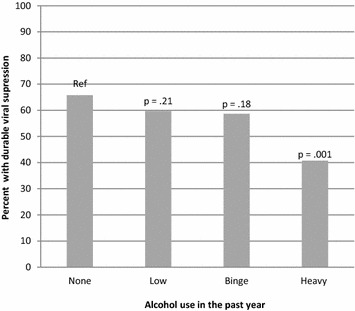
Proportion of 619 persons with HIV with durable HIV viral suppression (HIV viral load undetectable or <200 copies/ml at each test in the past year). Binge drinking defined as exceeding daily (but not weekly) drinking recommendations, and heavy drinking defined as exceeding weekly drinking recommendations. Florida Cohort, 2014–2016

The proportion of persons with optimal ART adherence (≥95%) was significantly lower with each increasing category of alcohol consumption, compared to those who were abstinent (Fig. 1). Specifically, optimal ART adherence was reported by 80% of non-drinkers, 68% of low level drinkers, 58% of binge drinkers, and 51% of heavy drinkers. In contrast, durable viral suppression was significantly lower only in persons with heavy drinking (41%) compared to those who were abstinent (66%), but there was no significant difference in durable viral suppression in persons with low-level drinking (60%) or binge drinking alone (59%) compared to those who were abstinent (Fig. 2). In multivariable analyses, heavy drinking, compared to abstinence, was associated with suboptimal HIV viral load suppression in both crude analyses (OR 2.79; 95% CI 1.50–5.21) and adjusted analyses (OR 2.16; 95% CI 1.08–4.32) (Table 3). In contrast, binge drinking alone (without heavy drinking) was not associated with a significant change in overall rates of optimal viral suppression (unadjusted OR 1.35, 95% CI 0.87–2.10; adjusted OR 1.04, 95% CI 0.64–1.70) (Table 3).

**Table 3:** Crude and adjusted associations between alcohol consumption and lack of durable viral load suppression among PLWH in the Florida Cohort (N = 619), 2014–2016

Characteristic	Crude estimates			Adjusted estimates		
	OR	95% CI	p value	AOR	95% CI	p value
Gender (ref = male)						
Female	0.83	0.60–1.17	.29	0.72	0.49–1.06	.09
Race (ref = Non-Hispanic White)						
Non-Hispanic Black	2.04	1.34–3.10	.69	1.85	1.17–2.94	.009
Hispanic	1.12	0.65	<.001	1.08	0.60	.80
Other	0.93	0.36	.88	0.71	0.26	.51
Age group (ref ≥ 55)						
18–34	3.77	2.18–6.51	<.001	3.99	2.21–7.20	<.001
35–44	2.95	1.78–4.87	<.001	2.80	1.64–4.76	<.001
45–54	1.82	1.17–2.84	.007	1.69	1.06–2.69	.03
Education (ref > high school)						
<High school	2.11	1.42–3.14	<.001	1.79	1.13–2.84	.01
High school or equivalent	1.74	1.16–2.60	.007	1.48	0.95–2.31	.09
Homelessness (ref = no)						
Yes	2.38	1.53–3.71	<.001	1.87	1.14–3.07	.01
Current smoking (ref = no)						
Yes	2.00	1.43–2.80	<.001	1.46	0.99–2.16	.06
Illicit drug use (ref = no)						
Yes	1.46	1.04–2.06	.03	1.17	0.78–1.75	.44
Depressive symptoms (ref = no)						
Yes	1.02	0.72–1.45	.92	–	–	–
Anxiety symptoms (ref = no)						
Yes	1.27	0.89–1.81	.19	–	–	–
Drinking status (ref = abstinence)						
Low drinking	1.29	0.86–1.94	.21	1.19	0.76–1.88	.45
Binge drinking only	1.35	0.87–2.10	.18	1.04	0.64–1.70	.87
Heavy drinking	2.79	1.50–5.21	.001	2.16	1.08–4.32	.03

*PLWH* persons living with HIV, *OR* odds ratio, *AOR* adjusted odds ratio, *CI* confidence limits

In the mediation analysis, the majority of the relationship between heavy drinking and suboptimal HIV viral suppression was via a direct effect (RR 1.61, 95% CI 1.16, 2.05), with a smaller yet significant indirect effect mediated via poor ART adherence (RR 1.05, 95% CI 1.00, 1.13) (Table 4). Although the overall effect of binge drinking on suboptimal HIV viral suppression was not significant (Table 4), the data suggest that indirect effects (mediated by poor ART adherence) could be more important when considering the relationship of binge drinking to suboptimal HIV viral suppression.

**Table 4:** Direct and indirect risk ratios for suboptimal HIV viral suppression for various alcohol consumption patterns

	Low drinking	Binge only	Heavy drinking
Direct effect	0.97 (0.76, 1.20)	1.02 (0.79, 1.26)	1.61 (1.16, 2.05)*
Indirect effect	1.00 (0.97, 1.03)	1.04 (1.01, 1.10)*	1.05 (1.00, 1.13)*
Total effect	0.97 (0.75, 1.20)	1.07 (0.82, 1.32)	1.70 (1.24, 2.18)*

Florida Cohort (N = 619), 2014–2016

* p < .05. The indirect effect represents the effect mediated by ART adherence

## Discussion

In this sample of persons living with HIV infection in Florida, heavy alcohol consumption was associated with approximately twice the odds of having suboptimal HIV viral suppression compared to non-drinkers, even when accounting for several potential confounding variables. In contrast, adults who reported occasional binge drinking but did not meet criteria for heavy drinking were not as a group significantly different from non-drinkers in terms of overall durable HIV viral suppression. The findings demonstrate that differences in the way that alcohol consumption is measured and defined can result in different conclusions about the relationship of alcohol consumption with HIV-related health outcomes or behaviors.

Heavy drinking was also significantly associated with suboptimal ART adherence, so we assessed whether the association of heavy drinking to suboptimal HIV viral suppression was mediated by poor ART adherence. We found that only a small, albeit significant, proportion of the association was via an indirect (mediation) pathway. These findings suggest that much of the relationship between heavy drinking and suboptimal HIV viral suppression maybe due to other mechanisms such as direct biological effects of alcohol on the immune system or to the effects of alcohol on behavior, including engagement in care and persistence on ART therapy [31].

Approximately a quarter of the population met criteria for binge drinking but not for heavy drinking, and given that binge drinking was associated with suboptimal ART adherence, it was somewhat surprising that binge drinking was not also associated with poor HIV viral suppression. It could be that current ART medications are more forgiving in terms of ART adherence [32], or there could be other unmeasured differences between the heavy drinkers and binge drinkers in this sample. Our findings are also consistent with other research that has generally found the highest levels of alcohol consumption, but not all alcohol use patterns, to be associated with poor HIV viral suppression [9–11].

The evidence for a causal relationship between alcohol consumption and poor HIV viral suppression is supported by longitudinal data showing that increases in drinking correlate with worse viral suppression [9], and some evidence that a reduction in alcohol consumption is associated with improved HIV viral suppression [33]. However, several limitations of the current study warrant mention. Although we used an item from a validated scale [26], self-reported ART adherence tends to over-estimate adherence. We chose to use a traditional cut-point of 95% adherent to distinguish adequate from inadequate adherence, although viral suppression can be now achieved with lower adherence (e.g. 80%) with more recent ART regimens [32]. Our sample was a convenience sample of persons who had been in care during the previous year, thus may not represent the entire population of persons living with HIV. The proportion of persons who met criteria for heavy drinking (exceeding weekly drinking limits) was also somewhat lower than the proportion found in other samples of PLWH. We likely under-estimated actual consumption, in part, due to limited response options on our alcohol assessment instrument. Although we adjusted for several factors that could have represented confounding variables that were the true cause of poor outcomes, it is possible that some unmeasured variables were the true cause of poor viral suppression in the heavy drinkers. These limitations are balanced somewhat by our ability to obtain HIV viral suppression information from statewide HIV surveillance, and by the diversity of our sample across gender, race, and location.

Detectable HIV viremia is associated with HIV disease progression and transmission of HIV virus to others, providing both individual and public health reasons to try to improve this outcome. If alcohol consumption contributes to HIV treatment failure, then interventions to address heavy alcohol consumption should become more routine in HIV clinical and public health settings. Our findings help reinforce the potential benefits of screening and brief intervention for alcohol problems in HIV care settings [22], especially when persons are having difficulties maintaining consistent HIV viral suppression. Future research should also seek to identify and address other mechanisms, such as retention in care, that could be affected by heavy alcohol consumption. We also need better strategies to identify which specific drinkers are in greatest need of intervention, and to identify and demonstrate the best strategies to help persons to reduce drinking.

## Abbreviations

HIV: human immunodeficiency virus
ART: antiretroviral treatment
PLWH: persons living with HIV
